# Supplementary material for: Immune selection suppresses the emergence of drug resistance in malaria parasites but facilitates its spread
Source: PLoS Comput Biol. 2021 Jul 19;17(7):e1008577. doi: 10.1371/journal.pcbi.1008577 (PMC8321109; doi:10.1371/journal.pcbi.1008577)
Supplement: S1 Text — (PDF) [file pcbi.1008577.s001.pdf]

S1 Text: Supplemental methods and data

Immunity

Immunity is calculated at each discrete time step and is equivalent to the percentage of parasites of strain *j* that are killed by host responses. The design is similar to that in [1], although that work used continuous time. Components of immunity, i.e., the innate and adaptive responses, given as *I* and *A*, respectively, represent percent activation. Adaptive immunity is strain-specific. Its total effect is increased by cross-reactive adaptive immunity. Innate immunity is strain independent. Both innate and adaptive immunity are modulated by a constant representing killing power, k_i and k_a, respectively. Immune efficacy may be decreased by saturating immunity. Each of these elements will be explained in detail below, but the total immune killing, given as Z_j, that is experienced by strain *j* is calculated as:

$$Z_j(t) = S(t)(k_i I(t) + k_a(A_j(t) + C(t))), \tag{1}$$

where *I(t)* is innate immunity at time *t*, $A_j(t)$ is adaptive immunity against strain *j*, *C(t)* is cross reactive immunity from another strain, and *S(t)* represents immune saturation.

Adaptive immunity: Adaptive immunity is calculated independently for each strain at each time step, based the density of the strain and the duration of exposure to that strain in time steps. At time *t*, let $A_j(t)$ be adaptive immunity to strain *j*, $D_j(t)$ be density of infected RBCs of strain *j*, $E_j(t)$ be the duration of exposure to strain *j*, and $A_j(t+1)$ be adaptive immunity to strain *j* at time *t + 1*. If the host is currently infected with strain *j*, the change in adaptive immunity can be described as follows:

$$A_j(t+1) = g_a A_j(t)\frac{D_j(t)}{\zeta + D_j(t)} - \tau A_j(t)\left(1 - \frac{E_j(t)^\delta}{E_j(t)^\delta + \zeta^\delta}\right) \tag{2}$$

The first term describes the growth of the adaptive immune response, with the specific growth rate, g_a, a property of individual hosts. The second term is a decay term representing antigenic escape due to variant switching. The constant ζ is the exposure duration at which the decay effect is half of its maximum, δ governs the shape of the relationship between exposure duration and decay of immunity due to antigen escape, and τ is the decay of immunity due to antigenic escape. As duration of exposure increases, fewer novel antigenic variants remain and thus, the rate of switching slows and antigenic escape decreases

over time. These terms combined produce a pattern of dampened oscillations, in both immunity and parasite populations. Adaptive immunity rises in response to expansion of the parasite population, consequently causing a decline in parasite population. In response, density-dependent growth of the immune response slows, and antigenic escape becomes the driving force. As adaptive immunity falls, the parasite population expands again, thereby repeating the cycle. As exposure increases, the decay term contributes less until finally adaptive immunity only rises over time, eventually eliminating the parasite population.

If the host is no longer infected with the strain, it is simply a process of decay, described as:

$$A_j(t+1) = A_j(t) - \nu_a A_j(t) \tag{3}$$

Adaptive immunity is constrained between 0.001 and 1.

Next, we calculate the contribution of cross-reactive immunity to the total adaptive immunity experienced by strain *j*. Cross-reactivity is calculated from the non-self strain with the highest adaptive immunity, given here as strain *h*. Let $A_h(t)$ be adaptive immunity against strain *h* at time *t* and $C_j(t)$ be the cross-reactive immunity experienced by strain *j* at time *t*. The amount of cross-reactivity between strains is given as χ and total cross reactive immunity is capped by χ_{max}. The contribution of cross reactivity is as follows:

$$C_j(t) = \min(\chi_{\text{max}}, \chi A_h(t)) \tag{4}$$

Innate immunity: Changes in innate immunity are strictly density dependent and are strain independent. Let *I(t)* be host innate immunity at time *t*, *D(t)* be total infection density, *G* be innate growth and *H* be innate decay, so that innate immunity at time *t+1* is calculated as follows:

$$I(t+1) = I(t) + G(t) - H(t) \tag{5}$$

$$G(t) = \begin{cases} g_i D(t), & \text{if } g_i D(t) \leq 1 \\ 1, & \text{otherwise} \end{cases}$$

$$H_{(t)} = \begin{cases} \nu_i I(t), & \text{if } \nu_i I(t) \geq 0 \\ 0, & \text{otherwise} \end{cases}$$

As calculated, *I(t)* may exceed one, and that value will be used to calculate *I(t+1)*. However, the contribution of *I(t)* to total immunity is capped at one.

Saturating immunity: As the density of parasites in an infection increases, the immune system becomes overwhelmed, which is known as saturating immunity. As the infection density approaches the saturation threshold, the immune system's total killing power decreases [2]. At maximum saturation, we assume that the immune system's killing capacity is only 85% of its level of activation, a value given by α. The relationship between saturation and density is given as follows, where S is the efficacy of immune killing relative to its maximum value, *D(t)* is the total antigen density (here, assumed to include merozoites, infected RBCs, and gametocytes) at time t, and the constant η determines the shape of the relationship between density and saturation:

$$S(t+1) = \max(\alpha, 1 - \frac{D(t)}{\eta + D(t)}) \tag{6}$$

RBC lifecycle

In each time step, RBCs die and are replaced with p RBCs, with total number not to exceed RBC carrying capacity K. Uninfected RBCs are modeled as a pool, rather than individually, and so have no age. Let U stand for the uninfected RBC count and M represent infected RBC count. The dynamics of uninfected RBCs can be represented as follows:

In an uninfected host, background mortality removes a fraction m_u of RBCs.

$$U(t+1) = \min(U(t)(1 - m_u) + p, K) \tag{7}$$

In an infected host, uninfected RBCs are subject to bystander killing, given as β, with additional uninfected RBCs killed for each infected RBC, i.e,

$$U(t+1) = U(t) - \beta M(t) + p \tag{8}$$

The multiplier, β has been estimated to be between 1 and 19, and may change over the course of an infection [3–6]. Here, we use 8.75.

Parasite mortality

For each strain circulating within a host: at time t, let M_j represent infected RBCs of strain j, B_j represent the number of infected RBCs of strain j bursting, Q_j represent the number of newly infected RBCs, m_M represent the background mortality rate of infected RBCs, Z_j represent immune killing against that strain, and W_j represent RBCs infected with a sensitive parasite of strain j. The host's drug treatment status is given as V, with 1 representing a treated host and 0 an untreated host, and ω represents treatment efficacy for sensitive parasites. The number of infected RBCs of strain j one time step ahead is then given by:

$$M_j(t+1) = M_j(t) - B_j(t) - m_M M_j(t) - Z_j(t)M_j(t) - V(W_j(t)\omega) + Q_j(t) \tag{9}$$

We assume gametocytes share epitopes with parasites at other life stages, so gametocytes experience the same immune process as well as daily background mortality. Gametocytes are not targeted by drug treatment. We track the number of gametocytes of a given strain, L_j, over time with:

$$L_j(t+1) = L_j(t) - Z_j(t)L_j(t) - \gamma L_j(t) + B_j(t), \tag{10}$$

where γ is the daily fraction of gametocytes killed, $B_j(t)$ is the number of gametocytes of strain j maturing at time t, and $Z_j(t)$ is immune killing for strain j.

Due to stochasticity and variability in host immune responses, there is a wide distribution of primary infection duration, producing both chronic and acute infections (S1 Fig). In the first few time steps, which are equivalent to days, after an infection enters the blood stage, parasite growth is unrestrained by immunity and grows exponentially (S2 Fig). Innate immunity rises in response and quickly becomes fully activated, typically by day five (S2B Fig). Innate immunity is responsible for most of the initial decline in parasite density. Adaptive immunity rises more slowly (S2C Fig), with rate determined by an individual host's adaptive immune growth rate (g_a). Infections clear during the acute stage if adaptive immunity rises quickly enough to exert a significant impact before innate immune activation falls, as in host 1 (teal curves in S2 Fig). Long infections produce higher adaptive immunity as well as exhausting the potential for antigenic escape.

During the chronic infection stage, parasite densities oscillate over time, gradually declining as adaptive immunity builds. Adaptive immunity oscillates due to feedback between parasite density and antigenic escape. After the infection is cleared, adaptive immunity gradually decays.

Reinfection with the same strain produces low density infections of relatively long duration. Infections

can be established because adaptive immunity prevents the parasite population from rising to a density that would trigger strong innate activation (S3 Fig). A host with high adaptive immunity from a prior infection, as in host 2 (purple), substantially suppresses the infection and eliminates it more rapidly than a primary infection (S3A Fig). Infections in a host with low adaptive immunity from a prior infection can last as long as a primary infection because the short primary exposure leaves potential for antigenic escape. Infections remain subpatent throughout. Transmission is unlikely in either case–even though asexual replication is ongoing, high levels of immunity mean that most gametocytes will be killed during the maturation delay, before they are transmissible.

Reinfection with a novel strain produces a brief, dense, symptomatic infection (S3B Fig). The infection can grow rapidly due to the weaker adaptive immunity conferred by cross-reactivity, but the rapid parasite expansion also triggers the innate immune response, curtailing the infection. These infections are transmissible, albeit much less so than primary infections. Although adaptive immunity still kills most gametocytes before they become infectious, enough are produced that some survive to maturity.

While the model did not produce infections of intermediate length, we expect that this had little effect on population dynamics. Because adaptive immunity and rate of adaptive immune escape are functions of the duration of exposure, a different distribution of infection duration would have two possible consequences. First, it could decrease the variability in the level of immunity produced by the primary infection. This could, in turn, decrease the mean immunity in the population. However, we expect the impact from either would be minimal. In S2C Fig, we show the rise of adaptive immunity over time. Adaptive immunity begins reaching peaks shortly after the end of acute infections. Infections would typically be eliminated when immunity is at or near maximum, so infections of intermediate length would still result in a high level of immunity. The only difference would be that, upon reinfection with the same strain, antigenic escape would occur more readily after a short infection than a long infection. Regardless, even short infections produce a level of exposure sufficient to suppress the density of secondary infections (S3 Fig), so intermediate duration infections would not directly affect the proportion of symptomatic infections. Finally, in these simulations, transmission intensity was high enough that most hosts were repeatedly reinfected. This would weaken any impact of variation in duration of chronic infections.

Drug treatment rates

When the rate of drug treatment was increased from the default 30% rate, equilibrium prevalence (S6 Fig) and rate of resistance evolution (S7 Fig) both increased, but the results were qualitatively similar to the

default treatment rate. The highest treatment rate that could be sustained without eradicating malaria in any replicate simulation (out of 20) was 40%. Any replicates that were eradicated were excluded from analysis. At treatment rates greater than or equal to 60%, the one strain and 300 vector condition achieved a mean equilibrium resistance prevalence greater than our threshold for ubiquity (75%). For one strain and 1200 vectors, the minimum treatment rate for ubiquitous resistance was 70%. Notably, even with a 70% treatment rate, the prevalence of resistance in the one strain conditions was lower and less stable than the prevalence of resistance in the 30 strain conditions under the default treatment rate. Interestingly, with a treatment rate of 20%, the overall pattern was similar to higher treatment rates in all conditions except the 30 strain, 1200 vector condition. Resistance was substantially suppressed in this condition, despite a effective treatment rate equivalent to the one strain conditions (S8 Fig), indicating that strength of selection for resistance was not sufficient to overcome immune selection.

Because reinfection with a previously-exposed strain produced a low density infection, the majority of infections in the one strain populations were asymptomatic. Therefore, although the same proportion of symptomatic infections were treated within each treatment rate condition, a substantially lower proportion of all infections were treated in the one strain conditions than in the 30 strain conditions (S8A and S8B Fig). However, low density infections were almost never transmitted and, as such, had little direct contribution to evolution. In order to more adequately represent the effective treatment rate, we measured the proportion of all gametocytes in the population that were in a host undergoing treatment (S8C and S8D Fig). Although the effective treatment rate was still suppressed with one strain, the rates were more similar between strain conditions. This indicates that the lower equilibrium prevalence of resistance in one strain conditions was not due to differences in effective treatment rate.

The effect of recombination and strain mutation rate

Within the host, mutation is the only way to break down linkage disequilibrium between the strain locus and the resistance loci. Within the vector, sexual recombination also breaks down linkage disequilibrium. Recombination would be expected to be beneficial if linkage disequilibrium is primarily negative, or, in other words, if most genotypes are of intermediate fitness because resistance and antigenic novelty are rarely found on the same genome. On the other hand, recombination could also impede the spread of resistance if it destroys beneficial associations between resistance mutations and rare strains. In either case, the effect of recombination is expected to be greatest in high transmission regions due to the increased genetic diversity of infections. To test this hypothesis, we measured the time to evolution of

resistance without recombination at four strain mutation rates, the default of 1×10^{-5}, a higher rate that was equal to the genomic mutation rate (2.5×10^{-5}), and two lower rates (5×10^{-6} and 1×10^{-6}).

Recombination had no effect on T_{ubiq} at any strain mutation rate (S9 Fig). This can likely be attributed to patterns of within-host strain diversity. Although negative frequency-dependent selection produced high diversity across all hosts in the population, strain evenness was lower within individual hosts because strains underwent successive selective sweeps. As a result, typically one strain dominated at any given time. Because recombination takes place only between gametocytes from a single blood meal, the effective recombination rate was low. Compared to Fig 1E in the main text, fewer strains were found in representative blood meals than in the infections at large (S10 Fig). This is at least in part because the gametocyte population in a given blood meal was much smaller than the parasite population in the infection from which it was drawn. Instantaneous measures of the strain richness of an entire infection captured new, low-frequency variants that would likely lost due to drift within a few rounds of replication.

Additionally, the model parameters introduced bias that would be expected to reduce the effect of recombination. The short genome and simple genetic architecture both decreased the likelihood of linkage disequilibrium, and therefore weakened any potential impact from recombination [7]. Therefore, it is not clear that this result can be generalized to natural populations.

At lower strain mutation rates, transmission intensity has little impact on T_{ubiq}. In these conditions, T_{ubiq} was similar to T_{ubiq} with 300 vectors at the default strain mutation rate (S9 Fig). This can be explained by considering strain diversity patterns within the population (S11 Fig). Strain diversity and population immunity were relatively low with lower strain mutation rates, and further, were similar between transmission intensities. Within infected hosts, novel strain mutations were less frequent, decreasing the opportunity for immune selection. As a result, high transmission did not delay the evolution of resistance. At the higher strain mutation rate, the relationship between transmission intensities was qualitatively similar to the default strain mutation rate, indicating a contribution from immune selection.

References

1. Bushman M, Antia R, Udhayakumar V, de Roode JC. Within-host competition can delay evolution of drug resistance in malaria. PLOS Biol. 2018;16(8):e2005712. doi:10.1371/journal.pbio.2005712.

2. Metcalf CJE, Graham AL, Huijben S, Barclay VC, Long GH, Grenfell BT, et al. Partitioning regulatory mechanisms of within-host malaria dynamics using the effective propagation number. Science. 2011;333(6045):984–8. doi:10.1126/science.1204588.

3. Jakeman GN, Saul A, Collins WE. Anaemia of acute malaria infections in non-immune patients primarily results from destruction of uninfected erythrocytes. Parasitology. 1999;119:127–133.

4. Haydon DT, Matthews L, Timms R, Colegrave N. Top-down or bottom-up regulation of intra-host blood-stage malaria: do malaria parasites most resemble the dynamics of prey or predator? Proc R Soc B Biol Sci. 2002;doi:10.1098/rspb.2002.2203.

5. Metcalf CJE, Long GH, Mideo N, Forester JD, Bjørnstad ON, Graham AL. Revealing mechanisms underlying variation in malaria virulence: effective propagation and host control of uninfected red blood cell supply. J R Soc Interface. 2012;9(76):2804. doi:10.1098/RSIF.2012.0340.

6. Fonseca LL, Alezi HS, Moreno A, Barnwell JW, Galinski MR, Voit EO. Quantifying the removal of red blood cells in *Macaca mulatta* during a *Plasmodium coatneyi* infection. Malar J. 2016;15(1):410. doi:10.1186/s12936-016-1465-5.

7. Iles MM, Walters K, Cannings C. Recombination can evolve in large finite populations given selection on sufficient loci. Genetics. 2003;165(4):2249–2258.
