# Supplementary material for: Immune selection suppresses the emergence of drug resistance in malaria parasites but facilitates its spread
Source: PLoS Comput Biol. 2021 Jul 19;17(7):e1008577. doi: 10.1371/journal.pcbi.1008577 (PMC8321109; doi:10.1371/journal.pcbi.1008577)
Supplement: S1 Table — (PDF) [file pcbi.1008577.s002.pdf]

Table S1. Table of symbols used in the supplemental methods

Symbol	Value	Meaning
K	8500000	Maximum number of RBCs
m_u	0.025	Mortality rate of uninfected RBCs
m_M	0.2	Mortality rate of infected RBCs
U	varies	Count of all uninfected RBCs
M	varies	Count of all infected RBCs
p	370000	Daily RBC production up to K
β	8.75	Bystander killing per infected RBC
γ	0.51	Gametocyte daily background mortality percent
g_i	0.00003	Innate immunity growth rate per time step
ν_i	0.85	Innate immunity decay rate per time step
g_a	Pois(0.85)	Adaptive immunity growth rate (property of individual hosts)
ν_a	0.001	Adaptive immunity decay rate per time step
k_i	0.6	Innate immunity kill rate per time step
k_a	0.8	Adaptive immunity kill rate per time step
ζ	1000	Adaptive immunity shape constant
ι	0.001	Initial value for adaptive immunity
τ	0.15	Antigenic escape
φ	180	Antigenic shape constant
δ	8	Decay of adaptive immunity due to antigenic drift
η	10000	Saturation shape constant
α	0.85	Maximum immune efficacy when immunity is saturated
χ	0.5	Cross-reactivity between strains
χ_{max}	0.5	Maximum immune contribution from a cross-reactive strain
ϑ	2.4×10^{-7}	Infection rate per merozoite
ω	0.999	Drug efficacy as daily mortality rate for sensitive parasites
