# Supplementary material for: Immune selection suppresses the emergence of drug resistance in malaria parasites but facilitates its spread
Source: PLoS Comput Biol. 2021 Jul 19;17(7):e1008577. doi: 10.1371/journal.pcbi.1008577 (PMC8321109; doi:10.1371/journal.pcbi.1008577)
Supplement: S2 Table — (PDF) [file pcbi.1008577.s003.pdf]

Table S2. List of parameters and default values

**Parameter**	**Value**
Host population size	100
Minimum RBC count	2000000
Host maximum parasitemia	Pois(11%)
Maximum host lifespan	500 days
Host daily background mortality rate	0.001
Gametocyte maturation time	2 days
Merozoite burst size	10
Instantaneous merozite mortality	20%
Liver stage delay	7 days
Transmission investment	5%
Sporozoite burst size	5
In vector survival rate	1%
Number of sporozoites injected into host	Pois(12)
Number of genomic loci associated with fitness and resistance	2
Genomic mutation rate	0.000025 per locus per replication
Resistance mu relative growth rate	0.4
Strain mutation rate	0.00001 per replication
Vector lifespan	20 days
Feed frequency	3 days
Infected feed delay	1 day
Treatment rate of symptomatic hosts	0.3
Threshold density for symptomatic infection	5000 merozoites/μL
Treatment duration	3 days
