# Supplementary figures and images for: Immune selection suppresses the emergence of drug resistance in malaria parasites but facilitates its spread
Source: PLoS Comput Biol. 2021 Jul 19;17(7):e1008577. doi: 10.1371/journal.pcbi.1008577 (PMC8321109; doi:10.1371/journal.pcbi.1008577)

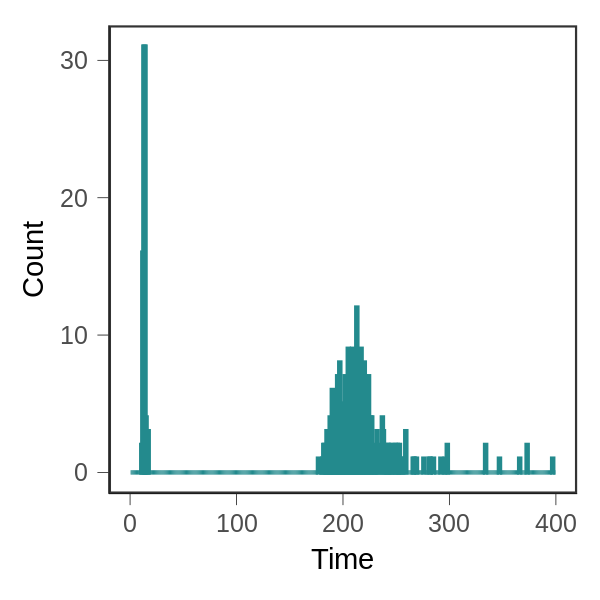

Supplement: S1 Fig — Infection clearance time was measured for 500 infections in untreated hosts. Approximately 50% of infections become chronic. Approximately 25% of infections have a rapid clearance time, represented in the early peak. Infections which result in mortality are not represented on this graph, but account for approximately 25% of primary infections. (TIF) [file pcbi.1008577.s004.tif]

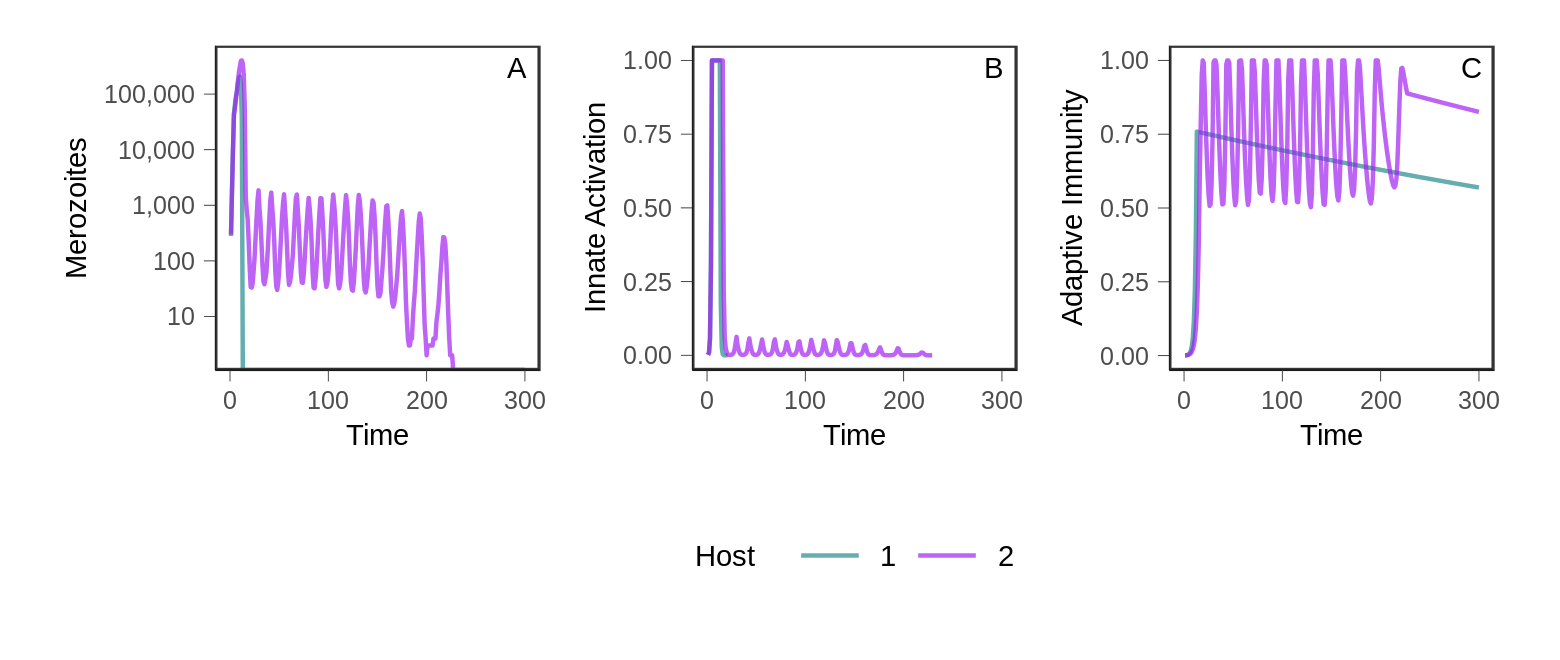

Supplement: S2 Fig — Two naive hosts are infected at time 0 with a 50 merozoites of a single strain. No mutation, treatment, or reinfection occurs. (A) Merozoite density/μL over time. Host 1 (teal) clears the infection rapidly. Host 2 (purple) shows the more common course of infection, under our default parameters. (B) Innate immunity over time. (C) Adaptive immunity over time. (TIF) [file pcbi.1008577.s005.tif]

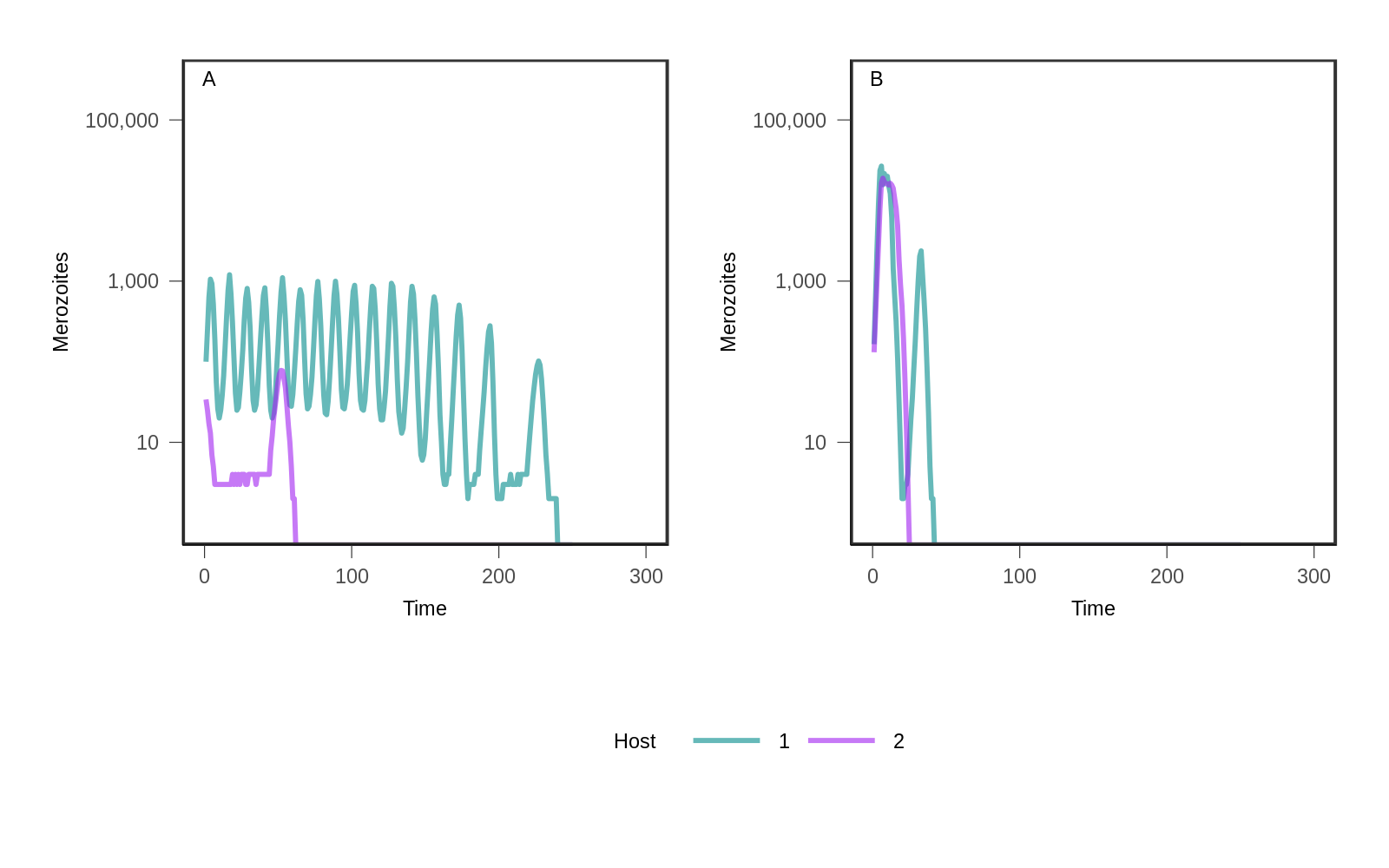

Supplement: S3 Fig — The hosts from S2 Fig were inoculated with 50 merozoites at time 300. (A) Reinfection with the same strain. (B) Infection with a novel strain. (TIF) [file pcbi.1008577.s006.tif]

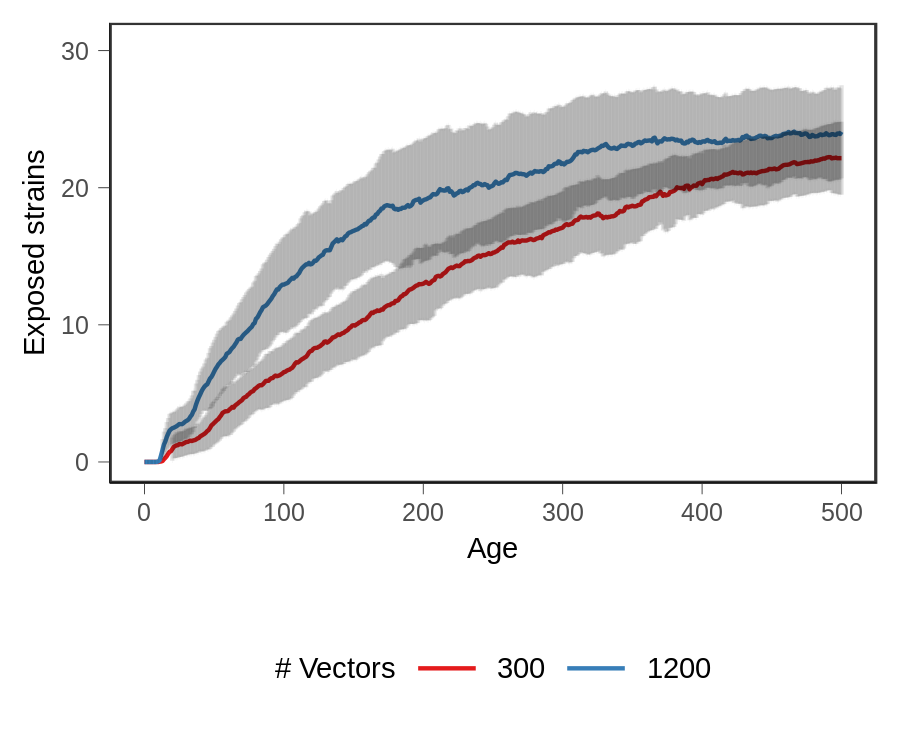

Supplement: S4 Fig — High transmission increased both the rate of exposure to new strains as well as the total number of strains to which hosts are ever exposed. Age is given in days. Values were calculated from 10 replicate untreated equilibrium populations. Lines represent means and shaded regions are standard deviations. (TIF) [file pcbi.1008577.s007.tif]

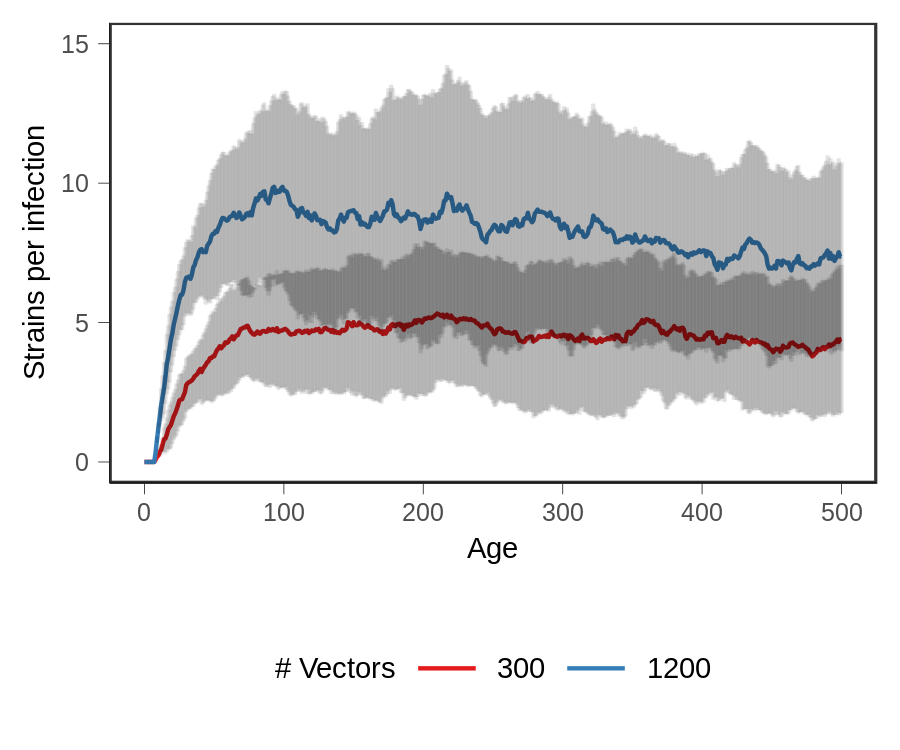

Supplement: S5 Fig — High transmission produced more complex infections at every age, reaching a peak at approximately 100 time steps. Even though older hosts had been exposed to more strains S4 Fig), complexity of infection did not increase after this peak, and in fact, slightly decreased with high transmission. Values were calculated from 10 untreated replicate equilibrium populations. Lines represent means and shaded regions are standard deviations. (TIF) [file pcbi.1008577.s008.tif]

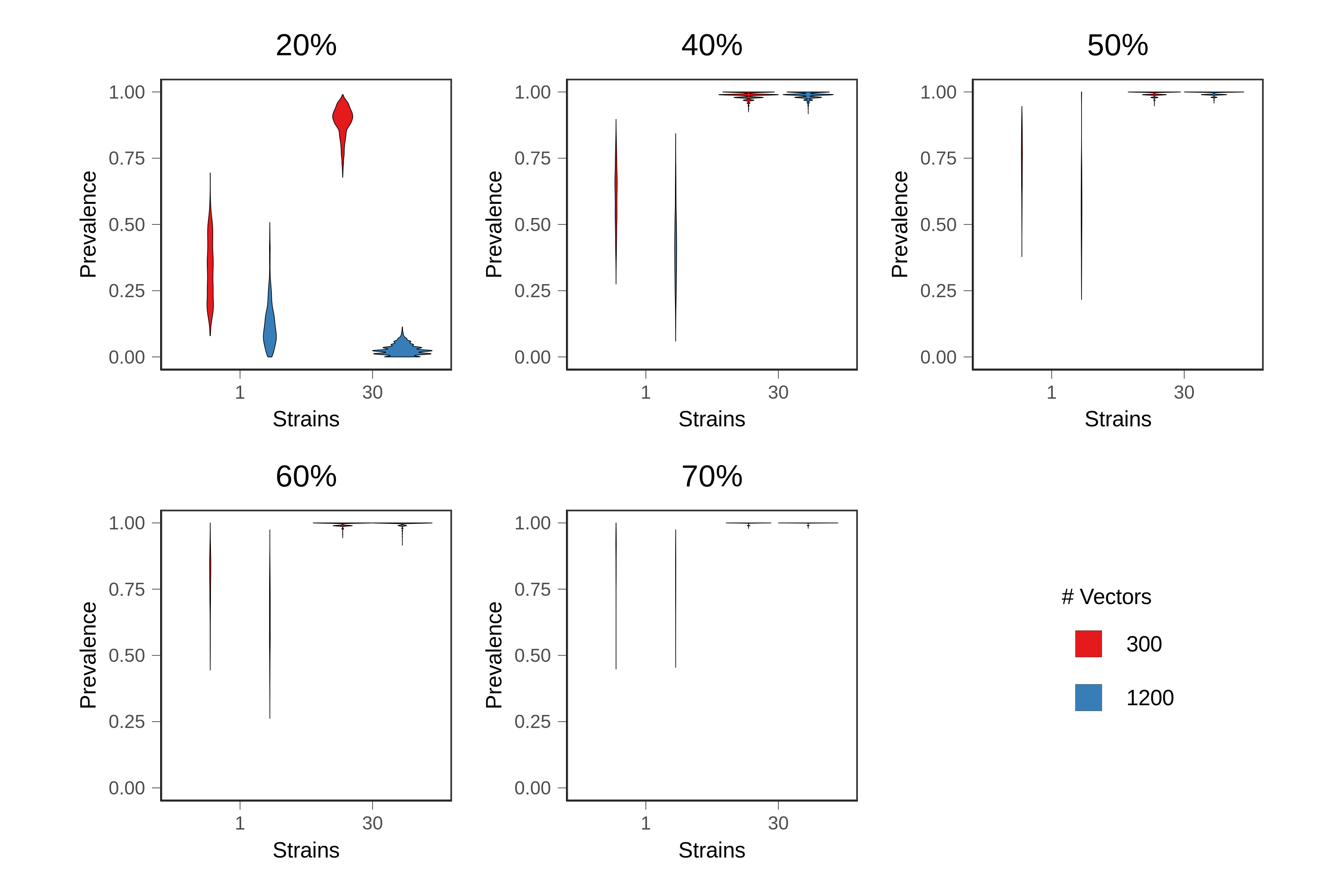

Supplement: S6 Fig — The equilibrium prevalence of treatment failure was measured in 20 replicate simulations, excluding replicates in which malaria was eradicated. Treatment rate for each simulation is indicated by the title of the graph. (TIF) [file pcbi.1008577.s009.tif]

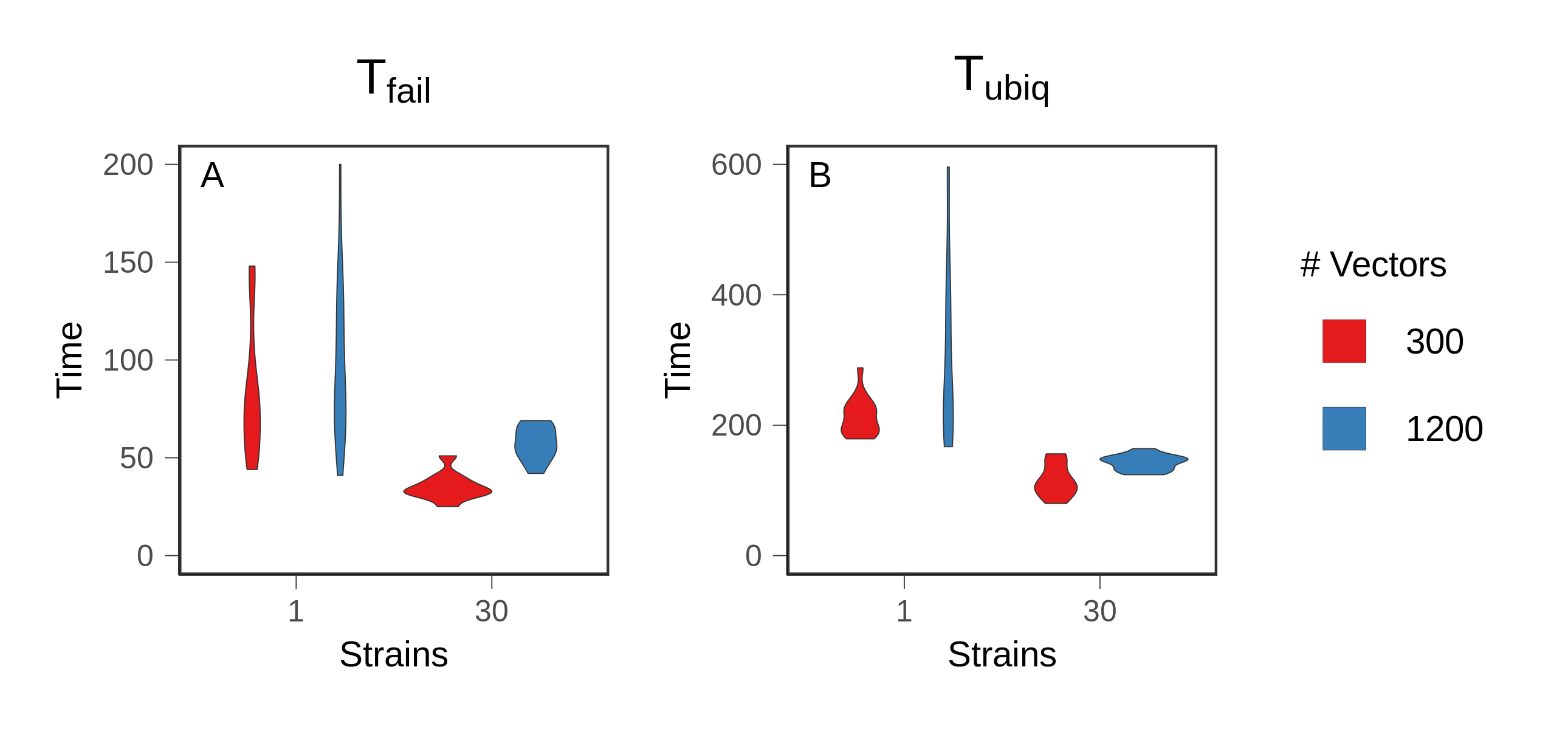

Supplement: S7 Fig — Mean time to resistance was measured over 20 replicate populations, excluding replicates in which malaria was eradicated. A: 10% prevalence of treatment failure (Tfail). B: 75% prevalence of treatment failure (Tubiq). Note different Y axes. (TIF) [file pcbi.1008577.s010.tif]

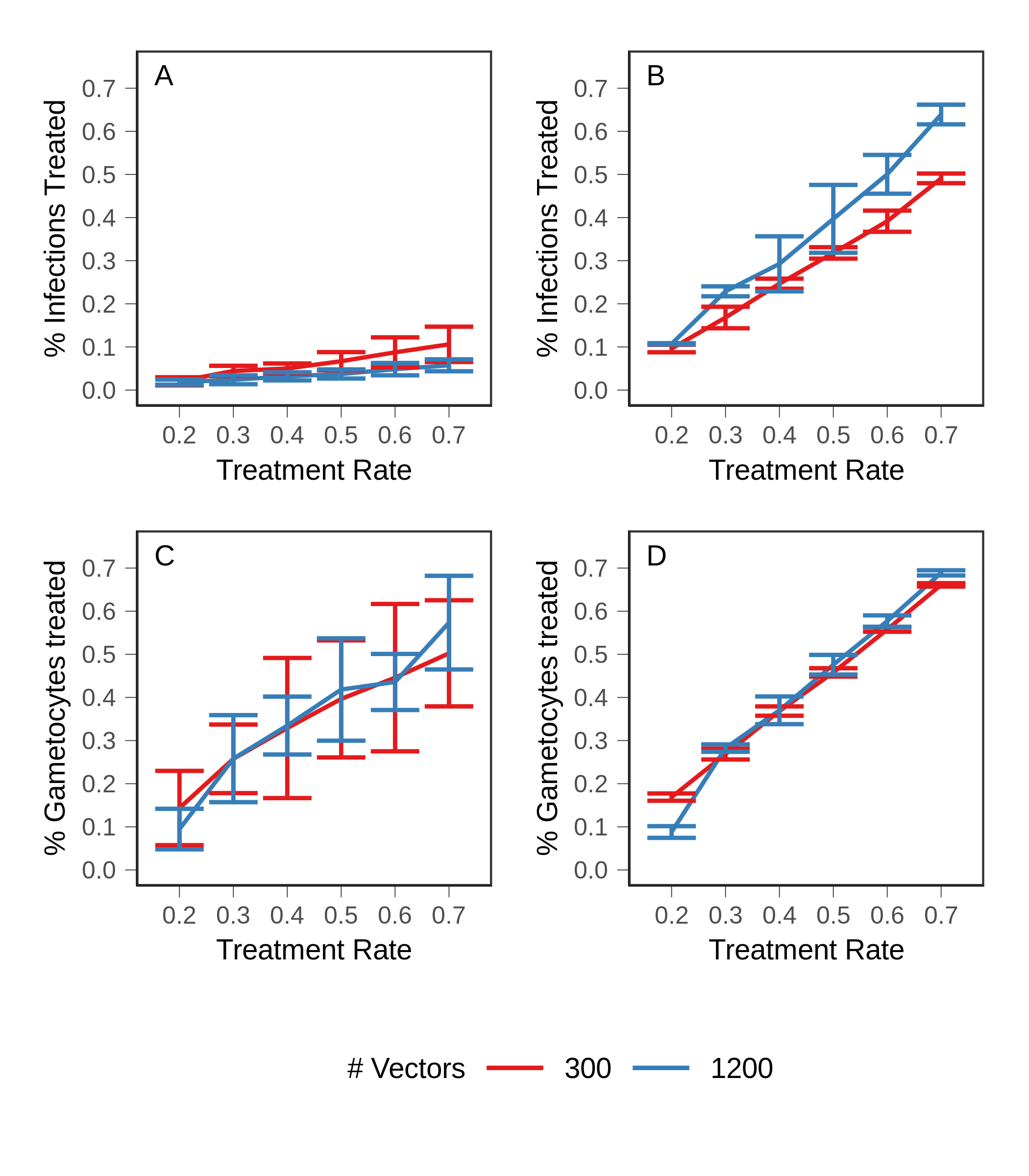

Supplement: S8 Fig — Effective treatment rates were measured for 20 replicate equilibrium populations over 250 time steps with means and standard deviations shown. Top row: The proportion of all infections that were treated. A: One strain, B: 30 strains. Bottom row: The proportion of total parasite population gametocytes that are in a treated host. C: One strain. D: 30 strains. (TIF) [file pcbi.1008577.s011.tif]

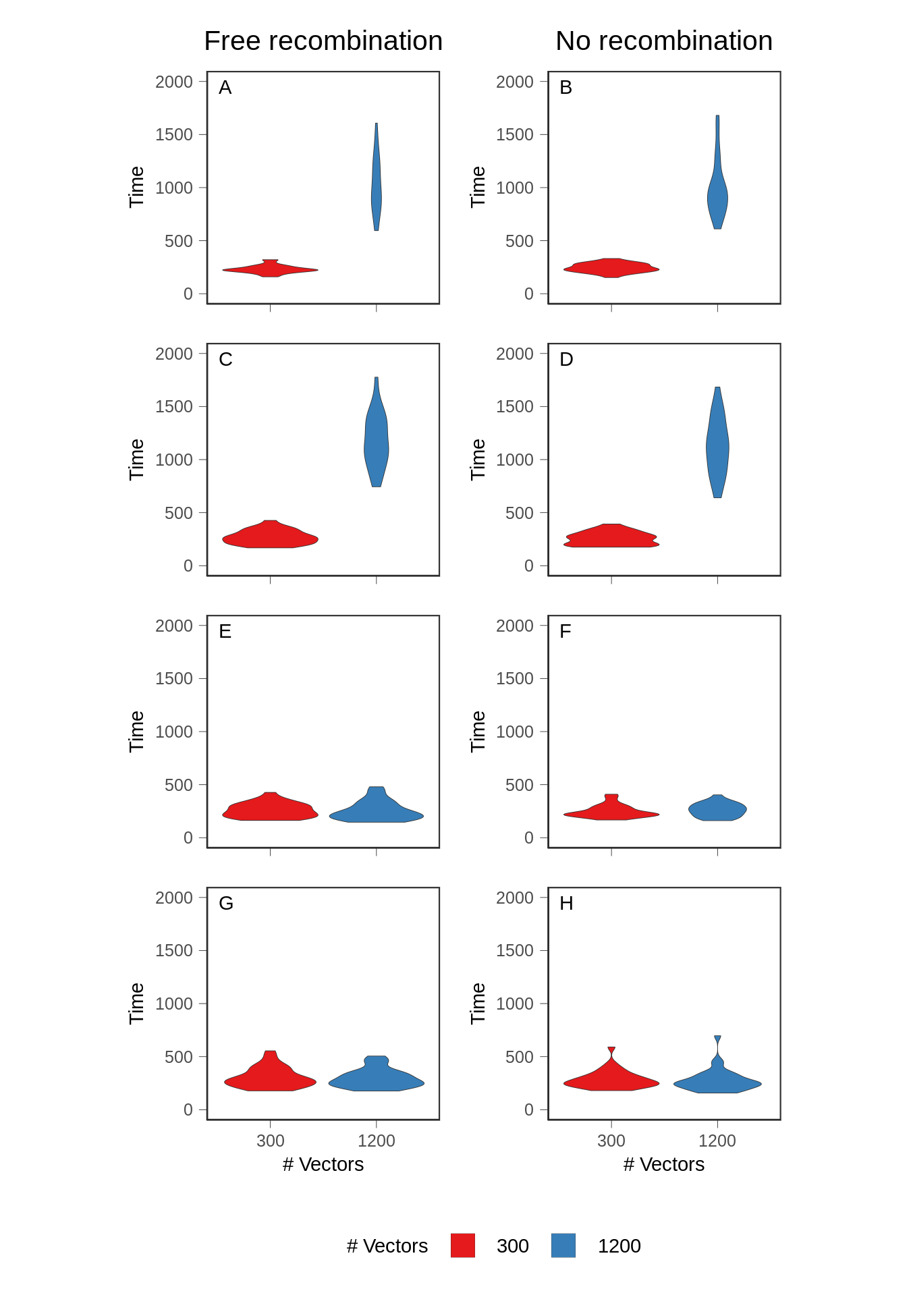

Supplement: S9 Fig — In populations with 30 strains, recombination between the strain locus and the resistance loci did not have a significant impact on evolution of resistance. Each row represents a different strain mutation rate. Top row: Higher strain mutation rate, equal to genomic mutation rate (2.5 × 10^-5^) (A) Tubiq with recombination. (B) Tubiq with no recombination. Second row: default strain mutation rate (1 × 10^-5^) (C) Tubiq with recombination (repeated from Fig 3B in the main text). (D) Tubiq with no recombination. Third row: Reduced strain mutation rate 5 × 10^-6^ (E) Tubiq with recombination. (F) Tubiq with no recombination. Bottom row: Reduced strain mutation rate 1 × 10^-6^ (G) Tubiq with recombination. (H) Tubiq with no recombination. Tubiq. (TIF) [file pcbi.1008577.s012.tif]

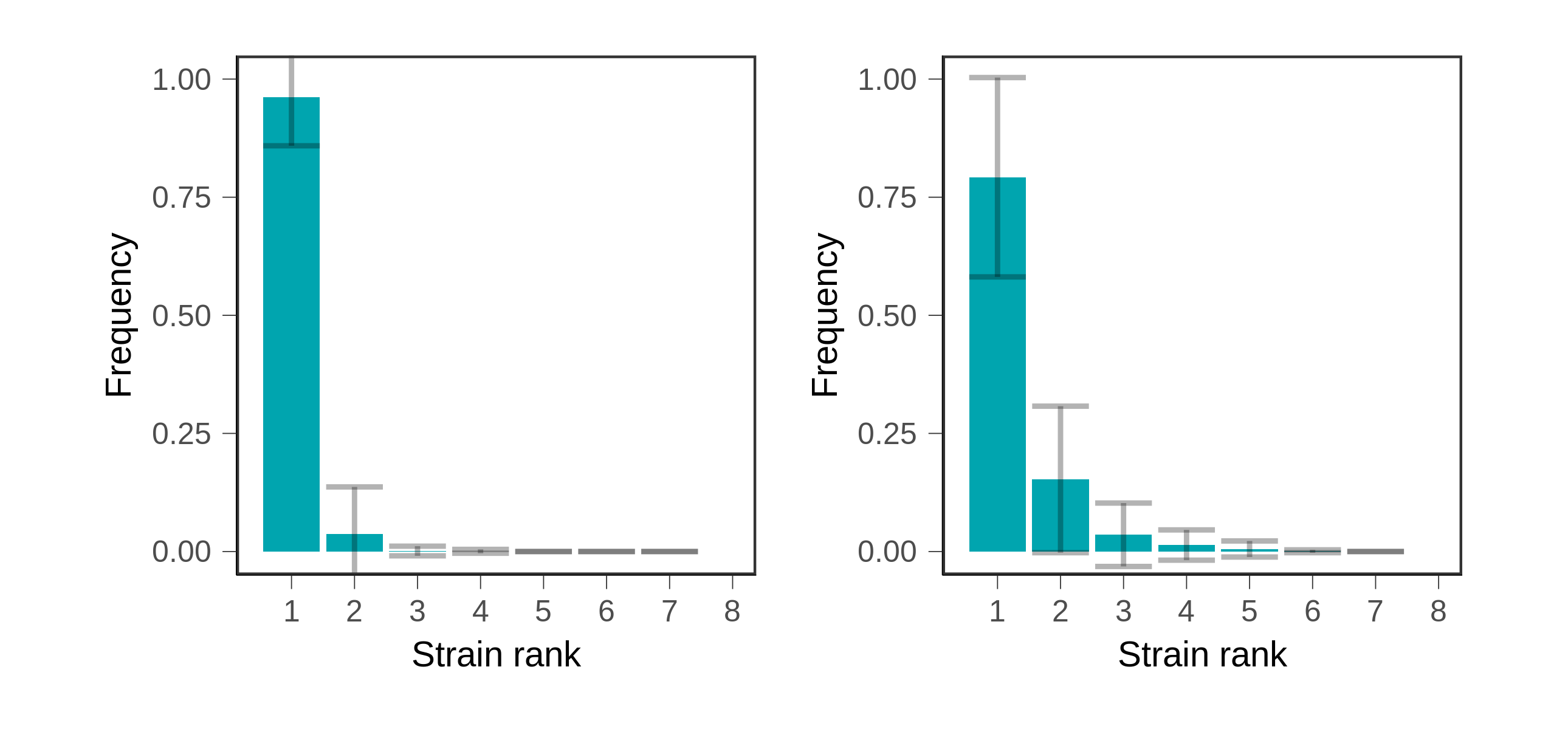

Supplement: S10 Fig — Representative blood meals were drawn from hosts in an equilibrium population and the frequencies of the strains it contained were ranked. With 300 vectors (left), most blood meals contained only a single strain, with minimal contribution from other strains. Diversity was higher with 1200 vectors (right), but the majority of the gametocytes within a single blood meal were still from a single strain. (TIF) [file pcbi.1008577.s013.tif]

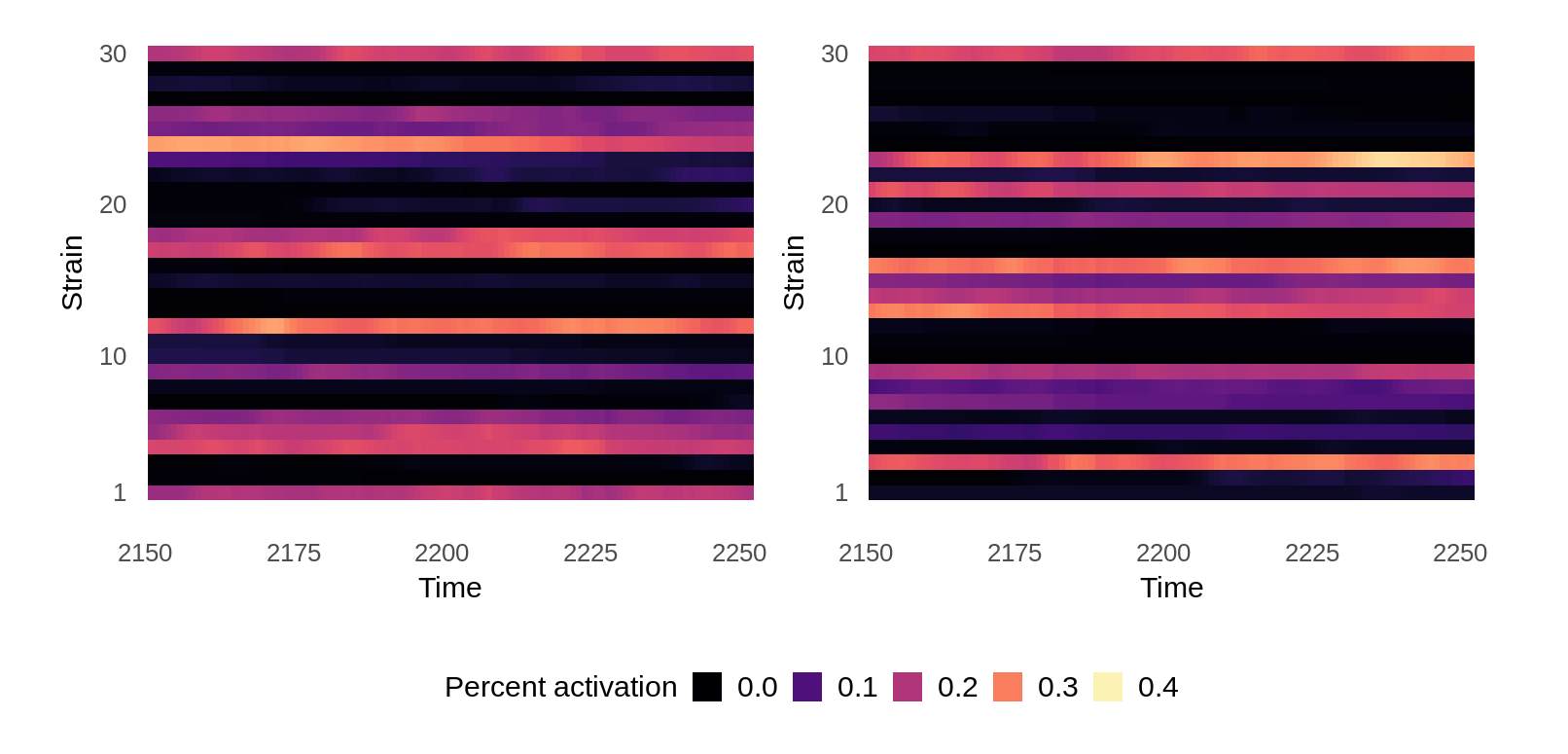

Supplement: S11 Fig — Populations were initiated with maximum strain diversity and then allowed to evolve to equilibrium over 2000 timesteps using a strain mutation rate of 1 × 10^-6^, an order of magnitude lower than the default. As in Fig 2 in the main text, immunity to each strain was averaged over all hosts in a single representative population. Strain diversity was lower with lower strain mutation rate and was similar between 300 vectors (left) and 1200 vectors (right). This demonstrates that lower strain mutation rates do not generate enough antigenic diversity to cause broad population immunity or induce immune selection. (TIF) [file pcbi.1008577.s014.tif]

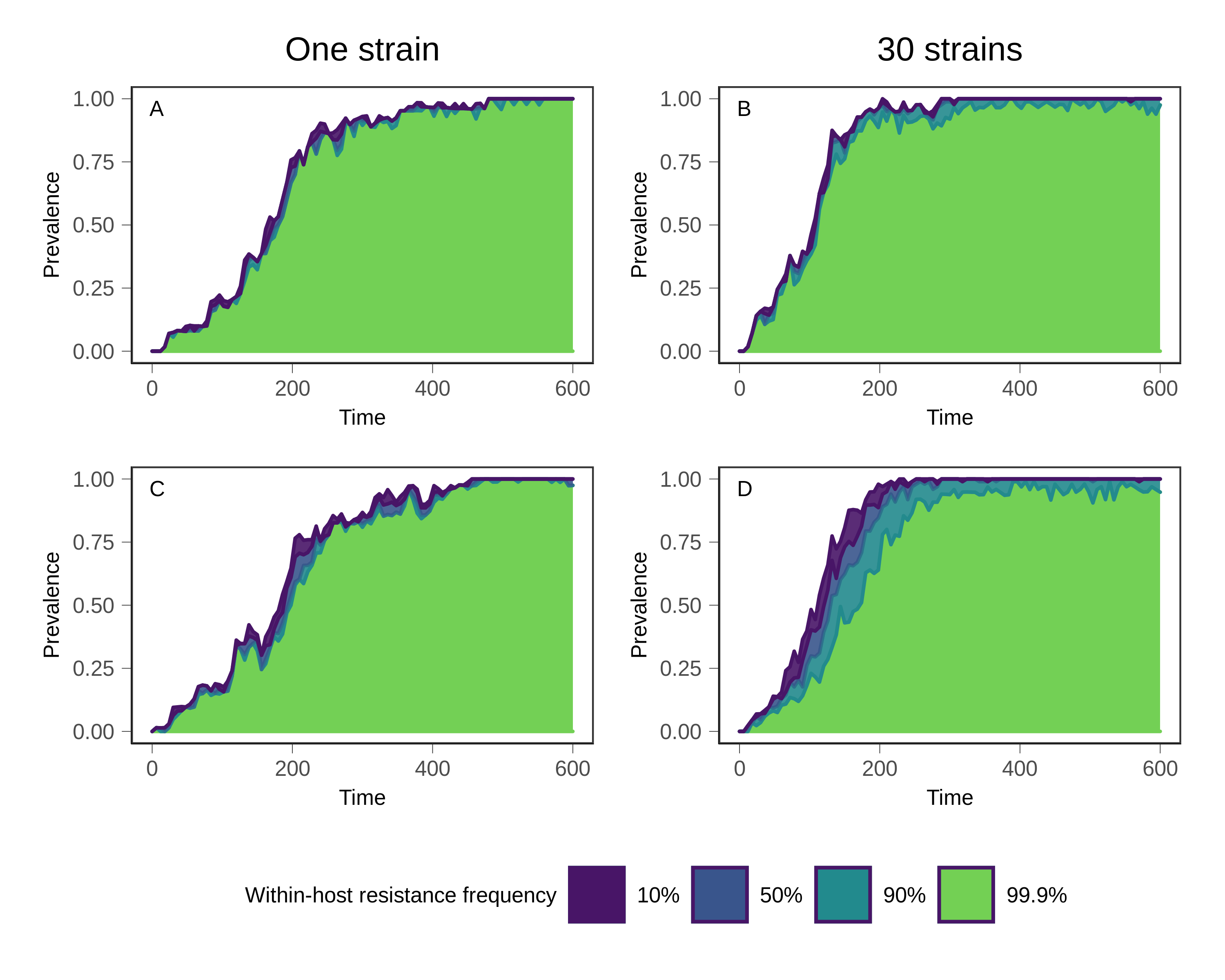

Supplement: S12 Fig — Following introduction of treatment at time 0, the prevalence of within-host frequencies of resistance was monitored over time. The top (purple) line indicates the prevalence of all treatment-resistant infections, defined as infections consisting of at least 10% resistant parasites. Moving down from the top, the prevalence of infections consisting of between 10% and 50% resistant parasites is shown in blue. Below that, the teal section shows the prevalence of infections in which the frequency of resistant parasites is between 50% and 90%. Finally, the bottom section in green shows the prevalence of infections in which resistance is at near fixation within the host, with a frequency exceeding 99.9%. Top row (A and B): 300 vectors. Bottom row (C and D): 1200 vectors. Left column (A and C): one strain. Right column (B and D): 30 strains. Patterns are qualitatively similar to costly resistance, in which mixed (i.e., sensitive and resistant) infections are rare within one strain conditions. Both 30 strain conditions show mixed infections at equilibrium, but, just as in costly resistance, mixed infections are common pre-ubiquity with 1200 vectors, indicating the greatest role for immune competition there. Data shows one representative simulation for each condition. (TIF) [file pcbi.1008577.s015.tif]

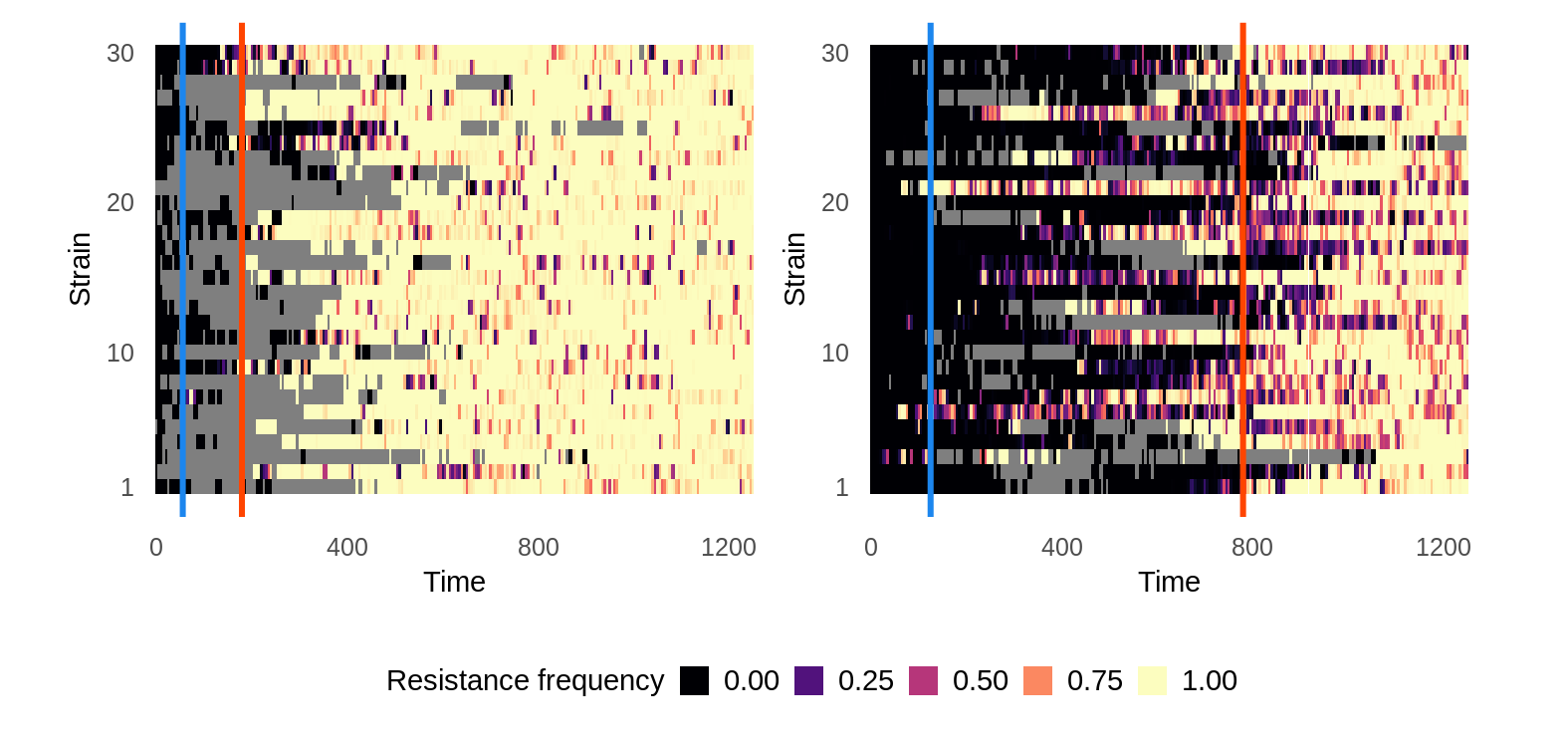

Supplement: S13 Fig — The frequency of resistance in each strain was monitored from treatment introduction to equilibrium. Left: 300 vectors. Most strains are fully resistant at equilibrium. Right: 1200 vectors. At equilibrium, partially resistant strains are common, suggesting influence from within-host competition. Representative populations are the same as those in Fig 6 of the main text. Gray indicates the strain is not present. The blue line indicates Tfail and the red line indicates Tubiq. (TIF) [file pcbi.1008577.s016.tif]

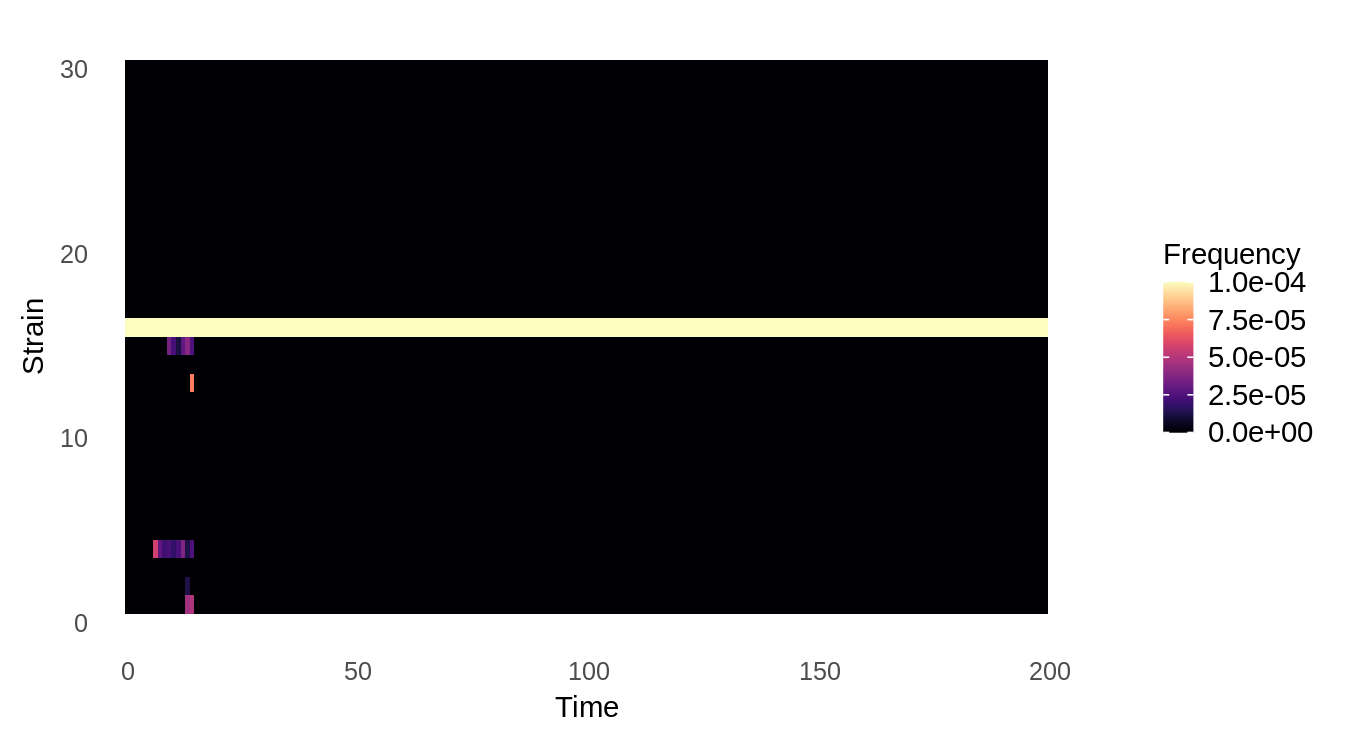

Supplement: S14 Fig — Every individual in a population of naive hosts was inoculated with parasites from a single strain. Strain mutation was permitted at the default rate, but cross reactivity between strains was 100%, so there was no selection for antigenic novelty. Strain mutants were produced during peak infection, but they remained at very low frequencies (note scale of the legend) and were quickly lost due to genetic drift. This demonstrates that strain diversity is almost entirely the result of selection. (TIF) [file pcbi.1008577.s017.tif]

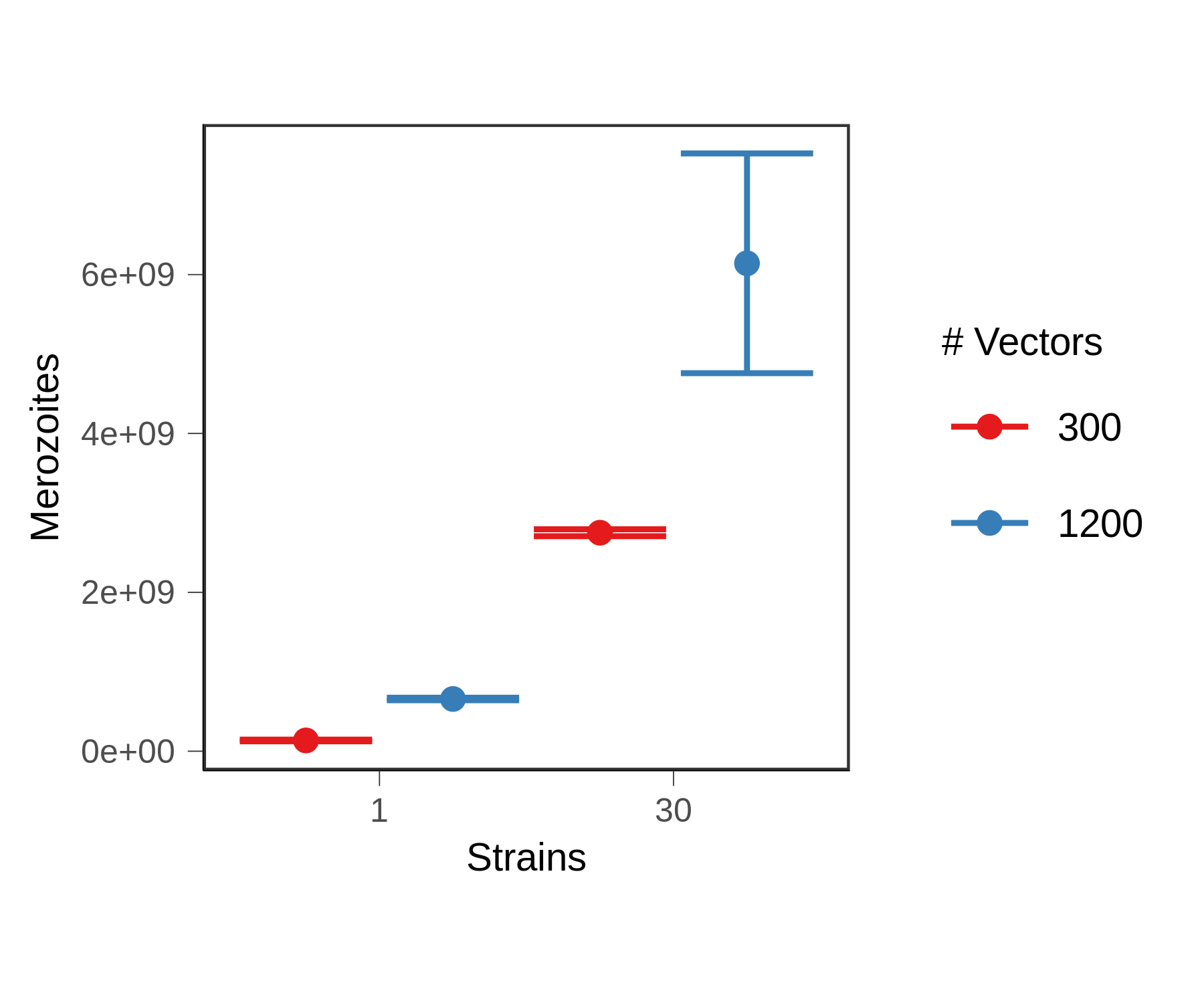

Supplement: S15 Fig — In order to determine if differences in mutation supply affected the origin of resistance, the density of all merozoites in ten replicate host populations was monitored for the first ten time steps after treatment was introduced. Despite the differences in population size, Tfail was similar between conditions (shown in Fig 3A in the main text), indicating that mutation supply is not a limiting factor. (TIF) [file pcbi.1008577.s018.tif]
